# Beyond the surface: a case report of penile tuberculosis as the first sign of miliary disease in a solid organ transplant recipient

**DOI:** 10.1128/asmcr.00141-25

**Published:** 2025-11-20

**Authors:** M. Tan, S. Green, C. Kenny, Y. O'Meara, S. Pisharoty, R. Hanson, G. J. Nason, G. Woods, F. Kelly, C. Mejia-Chew

**Affiliations:** 1 Department of Infectious Diseases, Mater Misericordiae University Hospital 8881, Dublin, Ireland; 2 Department of Nephrology, Mater Misericordiae University Hospital 8881, Dublin, Ireland; 3 Department of Plastic Surgery, Mater Misericordiae University Hospital 8881, Dublin, Ireland; 4 Department of Urology, Mater Misericordiae University Hospital 8881, Dublin, Ireland; 5 Department of Pathology, Mater Misericordiae University Hospital 8881, Dublin, Ireland; Pattern Bioscience, Austin, Texas, USA

**Keywords:** penile tuberculosis, miliary TB, transplant, SOT

## Abstract

**Background:**

Tuberculosis (TB) remains a major opportunistic infection in solid organ transplant recipients, with an incidence significantly higher than in the general population. Immunosuppression often leads to atypical and disseminated forms of TB, complicating timely diagnosis. Extrapulmonary TB can manifest in various genitourinary sites, but penile involvement is exceedingly rare, comprising less than 1% of urogenital TB cases.

**Case Summary:**

We report a confirmed case of penile TB in a 56-year-old abattoir worker with a history of renal transplant, who presented with a penile ulcer with cellulitis and systemic symptoms. Imaging revealed miliary pulmonary TB, confirmed on molecular testing from both respiratory and skin tissue samples. The patient was commenced on a four-drug anti-tuberculous regimen with dose modifications due to drug interactions with immunosuppressive medications. Clinical improvement with resolution of the penile lesion and systemic symptoms was noted.

**Conclusion:**

This case highlights the need for increased clinical suspicion for TB in immunosuppressed patients presenting with atypical genitourinary symptoms. Early recognition and prompt initiation of treatment, alongside careful adjustment of immunosuppression, are critical to achieving favorable outcomes. Given the rarity of penile TB and the complexity of its management in transplant recipients, this case contributes to the limited literature and underscores the importance of a multidisciplinary approach.

## INTRODUCTION

Tuberculosis (TB) remains an important opportunistic infection in solid organ transplant (SOT) recipients. A global review of 187 publications identified 2,082 cases of TB in SOT recipients—over 80% in kidney transplants—with a median TB incidence of 2.37% (1). Prevalence ranges from 0.5% to 6.4% in low-endemic regions, rising to 15.2% in high-burden settings (2–5). SOT recipients are estimated to have a 20- to 74-fold higher risk of developing active TB than the general population (4, 6).

Compared with immunocompetent hosts, TB in transplant recipients often presents atypically with subtle systemic symptoms, such as fever, night sweats, or weight loss, delaying clinical suspicion. Extrapulmonary and disseminated disease are also significantly more common in this population, with frequent paucibacillary presentations that complicate establishing a diagnosis (1, 7, 8).

Extrapulmonary TB represents 5–45% of the ten million global TB cases diagnosed annually (9–11). Genitourinary TB is an uncommon extrapulmonary manifestation, with penile involvement being exceedingly rare, comprising < 1% of urogenital TB cases (12). Penile TB may present with a myriad of clinical manifestations, including papules, nodules, plaques, or ulcerative lesions, often prompting consideration of sexually transmitted infections or malignancy (13–15). Precise data on the frequency of specific presentations are scarce; however, ulcerative lesions appear most commonly in the literature (16, 17).

We report a histologically and microbiologically confirmed case of penile TB in a renal transplant recipient with concurrent miliary pulmonary involvement. This case illustrates the diagnostic complexity of penile TB in SOT, particularly in non-endemic settings where clinical familiarity is limited.

## CASE PRESENTATION

A 56-year-old man, born and raised in rural Ireland, presented with a 1-week history of progressively worsening penile pain, swelling, and erythema, prompting admission under the urology team. His past medical history was significant for cadaveric renal transplant 15 years prior for end-stage renal disease secondary to focal segmental glomerulosclerosis, on maintenance immunosuppression comprising tacrolimus 2 mg twice daily, mycophenolate 360 mg twice daily, and prednisolone 5 mg once daily. Additional comorbidities included hypertension, hypercholesterolemia, and monoclonal gammopathy of undetermined significance.

**Fig 1:**
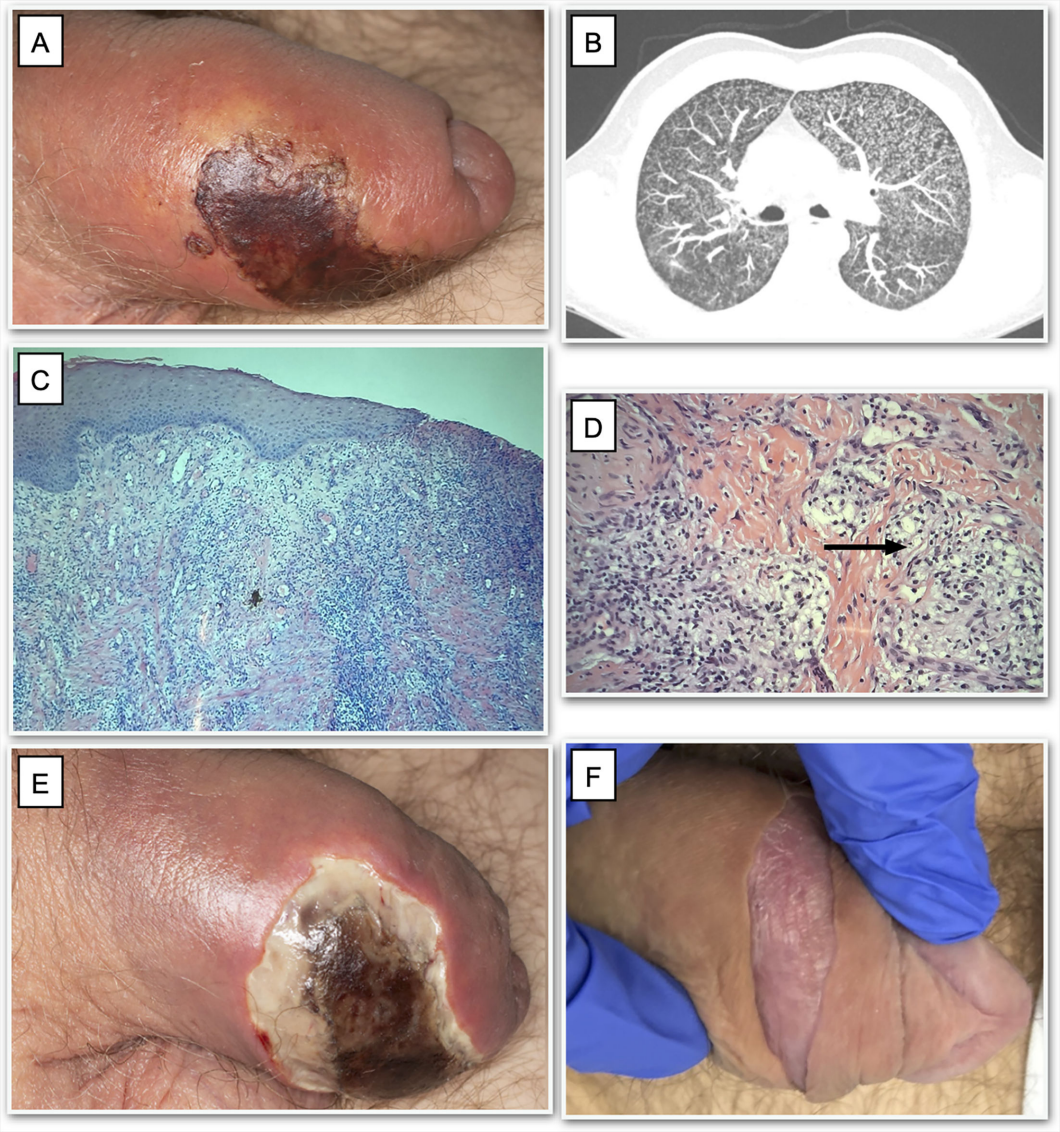
Penile ulcer, imaging, and histology. Penile ulcer on presentation (**A**). Computed tomography of the chest choosing miliary pattern (**B**). Punch biopsy showing ulceration on low power image using H&E stain (**C**). Non-caseating granulomas (black arrow) on high power image using Ziehl-Neelsen stain (**D**). Penile ulcer 10 days into anti-tuberculosis (TB) treatment (**E**). Penile ulcer 10 months into anti-TB treatment (**F**).

On admission, he was febrile (38.1°C) but hemodynamically stable. Genital examination revealed an indurated, erythematous area on the right posterolateral aspect of the penile foreskin, without scrotal edema or inguinal lymphadenopathy (Fig. 1A). The remainder of the examination was unremarkable. Laboratory investigations showed hemoglobin 11.9 g/dL (reference range: 13–18 g/dL), white cell count 12.41 × 10^9^/L (reference range: 3.5–11 × 10^9^/L), and C-reactive protein 62 mg/L (reference range: <7 mg/L). Renal function was at his usual baseline, with creatinine 130–150 µmol/L (reference range: 65–107 µmol/L) and estimated glomerular filtration rate of 45–55 mL/min/1.73 m² (normal range: ≥90 mL/min/1.73 m²). Liver function tests were within normal limits.

The patient was initially treated empirically for presumed penile cellulitis with intravenous piperacillin/tazobactam 4.5 g every 8 h and vancomycin 1.5 g every 12 h. Progressive swelling and erythema despite broad-spectrum antibiotics prompted early infectious diseases consultation. A comprehensive review uncovered a 3-month history of constitutional symptoms, including drenching night sweats, fevers, chills, anorexia, and unintentional weight loss (7 kg). Further history revealed he was raised on a farm, worked as a butcher handling deer and occasionally cattle, and was an avid hunter who field-dressed game. He denied TB contacts, incarceration, or homelessness. He was in a monogamous relationship with his asymptomatic wife. Recent travel included a mountaineering trip to Bilbao, Northern Spain, 4 months prior, and similar excursions across Scotland and Spain over the past 5 years.

Given the systemic symptoms, a CT thorax/abdomen/pelvis was performed, revealing diffuse miliary nodules consistent with miliary TB (Fig. 1B). MRI of the penis showed non-specific foreskin thickening without any masses or abscesses. TB became the leading differential, which prompted extensive microbiological work-up. QuantiFERON-TB Gold interferon-gamma release assay was positive. MTB complex DNA was detected in sputum using the Cepheid Xpert MTB/RIF Ultra assay, but mycobacterial cultures from sputum and bronchoalveolar lavage were negative. Punch biopsy of the penile lesion revealed non-caseating granulomas with a single acid-fast bacillus on Ziehl-Neelsen staining (Fig. 1C and D). MTB complex DNA was also detected in fresh penile tissue using Xpert MTB/RIF Ultra at Great Ormond Street Hospital. Histology excluded other neoplastic, infectious, or inflammatory processes, confirming the diagnosis of extrapulmonary TB.

Molecular testing performed at the Irish Mycobacteria Reference Laboratory included the GenoType MTBC assay to speciate the *Mycobacterium tuberculosis* complex, and the GenoType MTBDRplus and MTBDRsl version 2.0 assays for detection of drug resistance. All assays were negative for MTBC DNA, likely reflecting low bacillary burden in the biopsy specimen. All other microbiological investigations—including blood and urine cultures (mycobacterial cultures incubated on MGIT for 8 weeks), HIV serology, STI screen (VDRL/TPPA, urine NAAT for Gonorrhea and Chlamydia), BK virus PCR, CMV PCR, and serologies for *Rickettsia* and *Coxiella*—were negative.

Empirical anti-tuberculous therapy (ATT) was initiated using a four-drug regimen: rifabutin 300 mg once daily, isoniazid 300 mg once daily with pyridoxine 50 mg daily, pyrazinamide 1,500 mg once daily, and ethambutol 1,000 mg once daily. Rifabutin was chosen over rifampicin to minimize cytochrome P450-mediated interactions with tacrolimus, alongside close therapeutic monitoring. Mycophenolate mofetil was temporarily withheld to reduce net immunosuppression. Despite ATT initiation, the penile lesion progressed to necrotic ulceration with eventual foreskin breakdown (Fig. 1E), necessitating conservative sharp debridement by plastic surgery.

At 10 months, the patient had complete resolution of constitutional symptoms, gradual weight gain, and marked improvement of the penile lesion (Fig. 1F). A 12-month treatment course was planned due to immunosuppression.

## DISCUSSION

This case highlights penile TB as a sentinel presentation for miliary TB in SOT recipients. Penile TB is extremely rare, with urogenital TB more commonly affecting kidneys, ureters, bladder, prostate, scrotum, seminal vesicles, and occasionally, the urethra (13, 17). Penile involvement may occur via hematogenous spread or direct inoculation. In this case, dissemination from pulmonary involvement evidenced by miliary nodules on CT was the most likely source; however, the patient’s occupational exposure to animal carcasses also raised the possibility of direct inoculation, analogous to Prosector’s wart (18).

To our knowledge, penile TB in disseminated/miliary disease has not been previously described. Prior reports include two Iranian patients with glans penile TB (including one renal transplant recipient) but without miliary features and four additional cases reported by Gupta et al., one involving a female partner with endometrial TB (14, 19). In our case, the partner had no symptoms. Notably, a UK case reported culture-proven penile TB followed by endometrial TB in his partner a year later; molecular typing confirmed matching isolates, suggesting potential sexual transmission and underscoring the need for partner evaluation (20).

Ulcerative penile TB can mimic various conditions ranging from infectious causes such as HSV, granuloma inguinale, leishmaniasis, schistosomiasis, and LGV, to non-infectious causes, like cancer and other granulomatous diseases (15), making histological and microbiological confirmation essential. While molecular techniques are increasingly recognized for their diagnostic value in extrapulmonary TB, including urogenital/penile forms, and for enabling timely treatment (21), in our case, it was the histology demonstrating granulomas and Ziehl-Neelsen staining that provided confirmation of penile TB.

Although we could not isolate the MTB complex, we considered the possibility of *M. bovis* infection given the patient’s occupational and recreational exposures to animal carcasses, raising the possibility of zoonotic transmission. TB reactivation from the transplanted kidney is another consideration, which is a known risk in SOT recipients, especially when donor LTBI is undetected (1).

Treating TB in transplant patients is challenging, requiring a delicate balance between maintaining immunosuppression and preventing graft rejection (22). Rifampicin, a potent cytochrome P450 3A4 inducer, markedly reduces tacrolimus levels, thereby increasing rejection risk, whereas rifabutin is a safer alternative that minimizes drug interactions (23). Monitoring immunosuppressant levels and renal function is essential. In a Korean cohort of 2,144 SOT recipients, those receiving rifampicin needed more frequent and higher-dose calcineurin inhibitor adjustments, with 30% discontinuing treatment due to interactions and switching to levofloxacin or rifabutin (24). Similarly, a Chinese study of 2,032 SOT recipients reported increased hepatotoxicity, reduced cyclosporine levels, and graft rejection among rifampicin users. Conversely, liver transplant recipients on rifabutin experienced no adverse events (25). A retrospective study of 31 SOT recipients found similar cure rates across rifampicin, rifabutin, and non-rifamycin regimens, but graft rejection occurred exclusively in the rifampicin group (26). Encouragingly, all published cases of penile TB responded well to anti-TB therapy with full recovery (19, 27, 28).

### Conclusion

While genitourinary TB is rare, penile TB in an immunosuppressed SOT recipient may signal disseminated disease. Our case exemplifies the diagnostic and management challenges of TB in immunocompromised hosts and reinforces the value of multidisciplinary management and clinical vigilance for atypical TB presentations.
